# Spectral and network investigation reveals distinct power and connectivity patterns between phasic and tonic REM sleep

**DOI:** 10.1093/sleep/zsaf133

**Published:** 2025-05-21

**Authors:** Tamir Avigdor, Laure Peter-Derex, Alyssa Ho, Katharina Schiller, Yingqi Wang, Chifaou Abdallah, Edouard Delaire, Kassem Jaber, Vojtech Travnicek, Christophe Grova, Birgit Frauscher

**Affiliations:** Analytical Neurophysiology Lab, McGill University, Montreal, Quebec, Canada; Multimodal Functional Imaging Lab, Biomedical Engineering Department, McGill University, Montreal, Quebec, Canada; Center for Sleep Medicine, Croix-Rousse Hospital, Hospices Civils de Lyon, Lyon, FranceLyon Neuroscience Research Center, PAM Team, INSERM U1028 / CNRS UMR 5292 / Lyon 1 University, Lyon, France; Analytical Neurophysiological Lab, Department of Neurology, Duke University, Durham, North Carolina, USA; Analytical Neurophysiology Lab, McGill University, Montreal, Quebec, Canada; Analytical Neurophysiology Lab, McGill University, Montreal, Quebec, Canada; Analytical Neurophysiology Lab, McGill University, Montreal, Quebec, Canada; Multimodal Functional Imaging Lab, Biomedical Engineering Department, McGill University, Montreal, Quebec, Canada; Multimodal Functional Imaging Lab, Department of Physics, PERFORM Center / School of Health, Concordia University, Montreal, Quebec, Canada; Analytical Neurophysiological Lab, Department of Neurology, Duke University, Durham, North Carolina, USA; Department of Biomedical Engineering. Duke Pratt School of Engineering, Durham, North Carolina, USA; Institute of Scientific Instruments, Czech Academy of Sciences, Brno, Czech Republic; International Clinical Research Center, St Anne’s University Hospital, Brno, Czech Republic; Multimodal Functional Imaging Lab, Biomedical Engineering Department, McGill University, Montreal, Quebec, Canada; Multimodal Functional Imaging Lab, Department of Physics, PERFORM Center / School of Health, Concordia University, Montreal, Quebec, Canada; Analytical Neurophysiology Lab, McGill University, Montreal, Quebec, Canada; Analytical Neurophysiological Lab, Department of Neurology, Duke University, Durham, North Carolina, USA; Department of Biomedical Engineering. Duke Pratt School of Engineering, Durham, North Carolina, USA

**Keywords:** REM, microstate, phasic REM, tonic REM, spectrum, connectivity

## Abstract

Although rapid eye movement (REM) sleep is often thought of as a singular state, it consists of two substates, phasic and tonic REM, defined by the presence (respectively absence) of bursts of rapid eye movements. These two substates have distinct EEG signatures and functional properties. However, whether they exhibit regional specificities remains unknown. Using intracranial EEG recordings from 31 patients, we analyzed expert-labeled segments from tonic and phasic REM and contrasted them with wakefulness segments. We assessed the spectral and connectivity content of these segments using Welch’s method to estimate power spectral density and the phase locking value to assess functional connectivity. Overall, we found a widespread power gradient between low and high frequencies (p < 0.05, Cohen’s *d* = 0.17 ± 0.20), with tonic REM being dominated by lower frequencies (p < 0.01, *d* = 0.18 ± 0.08), and phasic REM by higher frequencies (p < 0.01, *d* = 0.18 ± 0.19). However, some regions, such as the occipito-temporal areas as well as medial frontal regions, exhibit opposite trends. Connectivity was overall higher in all bands except in the low and high ripple frequency bands in most networks during tonic REM (p < 0.01, *d* = 0.08 ± 0.09) compared to phasic REM. Yet, functional connections involving the visual network were always stronger during phasic REM when compared to tonic REM. These findings highlight the spatiotemporal heterogeneity of REM sleep which is consistent with the concept of focal sleep in humans.

## Graphical Abstract

**Graphical Abstract:**
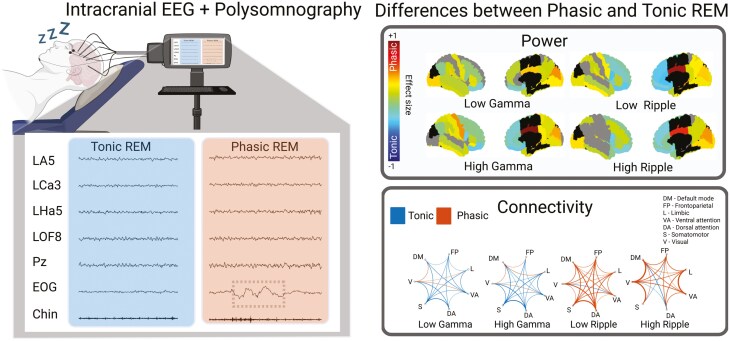

**Significance statement**

Phasic and tonic REM sleep display distinct and heterogeneous activation patterns depending on the region, network, and frequency band examined. Consistent with findings that sleep and wakefulness are local phenomena, we demonstrate that phasic and tonic REM also show region-specific electroencephalographic properties.

## Introduction

Rapid eye movement (REM) sleep, first described in 1953 [1, 2], is characterized by a desynchronized “wake-like” electroencephalogram (EEG), muscle atonia, and periods of rapid eye movements. REM sleep as a whole has been linked to vital roles such as brain maturation, synaptic regulation, emotional regulation [3], learning, and memory [4–6]. This state is often considered as homogeneous in research studies and sleep medicine, likely due to its characterization as such in the scoring rules that have dominated the understanding of sleep in recent decades [7]. However, REM sleep is not a uniform state but consists of periods with (phasic REM sleep) and without (tonic REM sleep) rapid eye movements. The difference between these substates has only recently become a focus of investigation [8].

The lack of granularity in the study of REM sleep is surprising, especially considering prior evidence demonstrating differences in information processing between phasic and tonic REM sleep. These differences are evident in a variety of cognitive domains such as arousal thresholds and sensory processing [9–13], attention [14], memory [6], dream recall [15] and dream content [16]. Previous research using scalp EEG suggests that tonic REM sleep may be an “in-between” state between phasic REM sleep and wakefulness. It thus becomes clear that REM sleep should not be treated as a homogeneous sleep stage, and that further investigation into human REM microarchitecture is needed [8]. The investigation of REM microstructure is particularly interesting in the context of high frequencies (> 30Hz), which have been linked to cognition [17], memory [18] and consciousness [19]. Since phasic and tonic REM display different attributes in these domains correlated with high-frequency activity, a comprehensive investigation of the high-frequency content of REM microstructure is warranted. Thus far, evidence from scalp EEG has hinted at the importance of high frequencies. For instance, some studies have used electrical source imaging to show that phasic REM sleep exhibits higher short-range connectivity within the low gamma (30–46Hz) band [20]. In addition, the same group also reported a gamma (31–48Hz) power increase in medial prefrontal and right lateralized temporal areas [21]. However, accurately measuring high frequencies using non-invasive methods is very challenging [22]. Additionally, electrical source imaging has limited spatial resolution, especially in deep-seated regions [23, 24], which is pivotal in the study of REM microstructure, as sleep has been shown to act both as a global and local phenomenon [25–28].

Stereo-electroencephalography (sEEG), used in the context of presurgical epilepsy evaluation, is a method of invasive intracranial EEG where electrodes are inserted into the brain. Given its high spatial and temporal resolution, it offers the unique possibility to accurately assess activity at the local spatial level, allowing careful investigation of high-frequency components, since it is less sensitive to muscle artifacts than scalp recordings. To date, investigations of human REM sleep substates using intracranial EEG have been sparse [8], as intracranial EEGs are only available in the context of epilepsy presurgical evaluations. As such, a large cohort of full-night recordings is required to obtain a whole-brain comprehensive full-spectrum investigation into the microstructure of physiological REM sleep. Despite this, even limited recordings in only a few patients with restricted spatial coverage have demonstrated the potential of this approach for studying high-frequency activity. For example, in six patients, it was shown that gamma activity (30–58 Hz) was higher during phasic REM than tonic REM in the neocortex [29]. In another six patients, gamma activity (40–70 Hz) in the orbito-frontal cortex was reported to be higher during phasic than tonic REM sleep [30]. In addition, in 7 patients, a decrease in alpha-low beta activity and an increase in high beta frequencies during phasic versus tonic REM sleep were observed in the primary motor cortex [31]. Finally, in 12 patients with thalamic sEEG recordings, it was also shown that thalamocortical phase connectivity was higher in the low gamma band (30–48Hz) during phasic REM compared to tonic REM [32]. However, to date, a whole-brain high spatiotemporal investigation into the spectral and connectivity differences between phasic and tonic REM has yet to be done. This is important to provide a complete understanding of the local differences across the spectrum between phasic and tonic REM, and how they compare to wakefulness. Exploring differences between phasic and tonic REM sleep with high spatial resolution across multiple brain regions has the potential to reveal local variations, similar to those previously demonstrated between wakefulness and sleep [27, 33]. Here, we conducted a fine-grained, systematic whole-brain, full-spectrum analysis of low and high frequencies using sEEG to assess, with high spatio-temporal resolution, the differences between phasic and tonic REM sleep compared to wakefulness.

Here, we conducted a systematic whole brain, full-spectrum analysis of low and high frequencies using sEEG to assess, with high spatio-temporal resolution, the differences between phasic and tonic REM sleep compared to wakefulness. We performed a within-patient, sleep cycle-controlled analysis of the electrophysiological properties of these three states of consciousness to determine how they differ. We hypothesize that: (1) There will be local differences between phasic and tonic REM compared to wakefulness, based on the concept of local sleep regulation; (2) High frequencies will display higher power and connectivity during phasic compared to tonic REM sleep, while lower frequencies will be stronger during tonic REM sleep, consistent with previous findings from studies with limited spatial coverage [31, 32]; and (3) varying trends in power and connectivity will be observed between wakefulness, phasic REM, and tonic REM, displaying distinct activation gradients across different regions and networks among the three states.

## Methods

### Patient and segment selection

**Figure 1.**
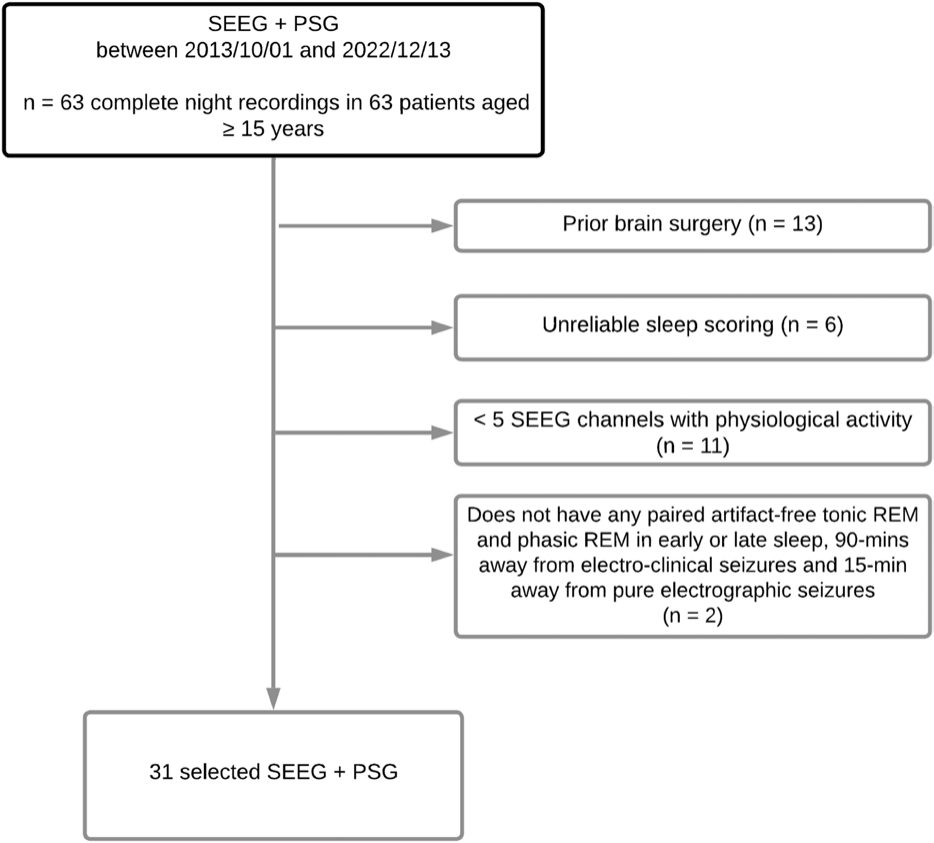
Patient selection flow chart.

We screened consecutive patients over 15 years of age with drug-resistant focal epilepsy who underwent sEEG recordings combined with simultaneous polysomnography (PSG), which included scalp EEG (3-9 channels), electro-oculogram (EOG), and chin electromyogram (EMG), for the scoring of sleep as part of their pre-surgical evaluation between 2013 and 2022. Based on the selection criteria, we included 31 patients (15 female; age = 36.7 ± 10.15 years) (see Flowchart in Figure 1). Inclusion criteria were: (1) patients with at least 5 non-epileptic channels outside the seizure-onset zone (SOZ), (2) presence of matching phasic and tonic REM periods with duration > 3 seconds, following a 90-minute seizure-free period to approximate one sleep cycle, (3) full-night recording and wakefulness from the prior day, (4) recordings sampled at 2000 Hz. Patients were excluded if they had (1) undergone prior surgery, (2) sleep scoring was not feasible, (3) absence of any clear artifact-free tonic and phasic REM with 3 second minimum duration that were at least 90 minutes away from electro-clinical seizures or 15 minutes away from electrographic seizures, and (4) absence of a 10-minute artifact-free wakefulness period more than 2 hours before sleep onset. The study was approved by the Montreal Neurological Institute and Hospital Review Ethics Board (00010120).

Sleep scoring was done by board-certified neurophysiologists (B.F. or L.P.-D) according to the American Academy of Sleep Medicine criteria [7]. The start and end of every sleep cycle was annotated. Both experts then independently marked artifact-free unambiguous phasic REM periods as burst of rapid eye movements detected on the EOG with a minimum duration of 3 seconds [34, 35], and unambiguous tonic segments featuring eye movement free periods during all REM sleep. Tonic and phasic segments were at least 5 seconds apart. This resulted in 1,626 phasic/tonic events with an average duration of 9.12s ± 7.17. After cross-checking the first fifteen phasic and tonic segments marked in each patient (or all segments if less segments were marked), the inter-rater agreement was found to be 97% for phasic and 98% for tonic segments. In total, a median of 49 phasic segments per patient (range: 9–179) was marked. Additionally, another neurophysiologist (C.A.) marked 10-30 seconds of artifact-free quiet wakefulness segments, resulting in a total of 10 minutes from the prior day between 2-4 hours prior to sleep onset. We then matched each phasic segment to the closest tonic segment of equal duration in the same REM sleep episode such that the phasic-tonic pair exhibited the same duration. Each part of the tonic signal was matched to only one phasic segment and was used only once. The tonic segments could be found either before or after the phasic segment, and the closest tonic segment, which was at least 5 seconds away from the selected phasic segment was chosen. To investigate differences, we performed paired comparisons between phasic and tonic REM sleep. Additionally, we compared both REM states to wakefulness data, which was selected from artifact-free segments of the previous day. Comparison between REM segments and wakefulness segments was completed in an unpaired manner, since they were not matched and durations were different. All comparisons were conducted at the channel level while controlling for the patient as a random effect.

### Recordings and channel classification

On average, 11.45 ± 3.15 [7–15] MNI sEEG electrodes (10 patients with 9 electrodes of 0.5–1 mm, and a distance of 5 mm between contacts) or DIXI sEEG electrodes (21 patients with 10–15 electrodes of 2 mm, and a distance of 1.5 mm between contacts) were used. Hardware filter settings were 0.1 Hz for the high-pass filter and 500 Hz (Stellate) or 600 Hz (Nihon Kohden) for the low-pass filter. The sampling frequency was 2000 Hz. A bipolar montage was employed for the scalp EEG (F3-C3; C3-P3; Fz-Cz; Cz-Pz; F4-C4; C4-P4) for the purpose of sleep scoring. Each sEEG channel underwent clinical assessment by an epileptologist (B.F.), and only channels considered normal, defined as containing no or only rare epileptic activity, were selected for further analysis, as done in our previous work [36, 37]. This selection process resulted in 1229 channels (39.64 ± 33.45 [8-159] channels per patient).

### Electrode co-registration and anatomical localization

The anatomical localization of electrodes was determined by co-registering preimplantation anatomical magnetic resonance imaging (MRI) data and post-implantation computed tomography or MRI data for each subject. Coordinates of individual channel contacts were then standardized in a common MNI space using minctools (http://bic-mni.github.io/) and the Intraoperative Brain Imaging System framework, as previously described [36, 37]. Channels were aggregated on a regional level using the reduced MICCAI atlas [36] composed of 38 regions without left-right distinction, and on a network level using the Yeo 7 networks atlas [38]. The MICCAI atlas was utilized for spectral analysis, and the Yeo 7 network atlas for connectivity analysis. Only anatomical regions or networks that had at least 5 channels in 3 patients were considered for further analysis (Figure 3).

### Analysis and metrics

The analysis of sEEG signals was carried out using a common average montage, which included all channels that did not contain epileptic spikes at a rate exceeding 3 spikes per 10 minutes. This approach was selected to mitigate the residual effects of epileptic activity [39] The signals were filtered using a Butterworth band-pass filter with a frequency range of 0.3 to 600 Hz and a 60 Hz notch filter.

We analyzed the fast frequencies bands (low gamma (30–50 Hz), high gamma (50–80 Hz), low ripple (80–140 Hz), high ripple (140–200 Hz) and very high ripples (200–500 Hz)), and then compared these results to those of traditional frequency bands (delta (0.5–4 Hz), theta (4–8 Hz), alpha (8–13 Hz), beta (13–30 Hz)) as well as functional bands (slow delta (0.5–2Hz), fast delta (2–4Hz), Iota (25–35Hz) [40] in order to further parse bands that are related to memory consolidation. Spectral analysis for every channel was performed using the Welch method, considering a 3-second window sliding with 50% overlap for each phasic REM, tonic REM, or wakefulness segment. We then applied within each region, paired comparisons between phasic and tonic segments, and unpaired comparisons between phasic or tonic REM segments and wakefulness segments. Functional connectivity analysis was assessed for each channel-pair using a phase-based measure: the phase locking value (PLV) [41] was computed in each frequency band separately as $PLV_{n,m,f}=\frac{1}{T}\left|\underset{t=1}{\overset{T}{\sum }}\;e^{-j\left(\phi_{n}\left(t\right)\;-\;\left(\emptyset_{m}(t\right)\right)}\right|$ where m,and   n are referring to channel indices, *f* is the frequency band of interest, *T* is the signal length, and ϕ is the instantaneous phase estimated as the angle of the analytical signal from the Hilbert transforms of both signals (i.e. from channels m and n). Only connections exhibiting at least low-moderate connections (defined as PLV > 0.1) were considered for further analyses. Connectivity was calculated either within a network, considering channel pairs located inside the same network, or between networks, considering channel pairs where one channel was located in one network and another channel in another network. We considered PLV as a phase-based metric of functional connectivity, as it has recently been shown to be effective in studying human sleep [42]. However, as in sEEG the amplitude of electrical potentials is decreasing in a quadratic manner from the distance to the generator, volume conduction is considered a more local phenomenon in sEEG when compared to scalp EEG, since recordings are located closer to the generators in sEEG [43]. Consequently, further distant channels may also exhibit zero phase lag that corresponds to a true phenomenon, rather than the result of volume conduction. Therefore, to examine the eventual perfect synchronicity, we opted to use the traditional phase-lag index without correcting for zero lag connections (0 and 360°) [44]. Nevertheless, we cannot exclude a potential small effect of volume conduction at the zero-lag. However, this was shown not to be only due to volume conduction [45].

### Statistical analysis

**Figure 2.**
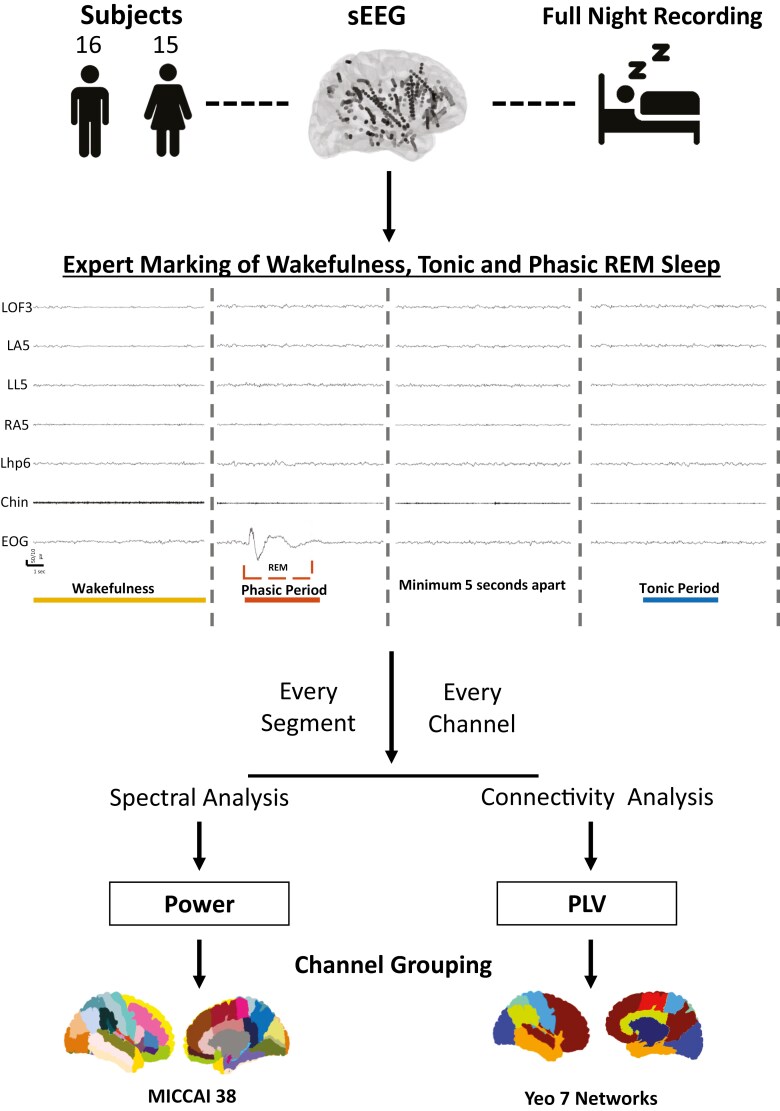
Schematic of the analysis pipeline. Patients with full-night sEEG recordings were analyzed. Experts marked wakefulness, tonic, and phasic REM segments. Displayed is a representative patient (#2) showing a few selected intracranial channels, chin electromyography (EMG), and electro-oculogram (EOG). EOG was used to identify rapid eye movements (see the dashed box). The duration of the selected segments is marked by the underline colored (yellow-wakefulness, phasic- red, tonic- blue). Segments were then analyzed for their spectral and connectivity content using Welch’s method and the phase locking value (PLV). Channels were then grouped into regions using the MICCAI 38, and the Yeo 7 network atlas respectively. Regions and networks with at least 3 patients and 5 channels were then tested for differences between tonic and phasic REM in a paired manner and then compared to wakefulness segments in a non-paired manner. LOF: Left orbitofrontal, LA: left amygdala, LL: left lingual gyrus, RA: right amygdala, Lhp: Left hippocampus.

For each available region, network, or network pair the estimated power or functional connectivity values were pulled together over all segments and all channels (Figure 2 ). We then weighted the power/PLV for each region to ensure that each patient’s contribution was equal regardless of the number of channels and phasic/tonic/wakefulness events used. Phasic and tonic REM had the same amount of events, while wakefulness consisted of varying numbers of 10- to 30-second events which totaled 10 minutes. Thus, the number of measures of phasic/tonic events or wakefulness events varied within a patient within a region/network/network-pair. For each patient p, the region r was measured using data from $N_{p,r\;}$ channels (or channel pairs for connectivity). We define the number of measurements associated to the region/network/network-pair r for the patient p as $S_{p,r}=N_{p,r\;\;\;}\times N_{event}$ where $N_{event}$ is the number of events measured for that patient (either phasic/tonic REM or number of wakefulness segments). The average number of measures for each region/network/network-pair ($\mu_{r}=\frac{\overset{P}{\underset{p=1}{\sum }}S_{p,r}\;}{P}\;$) was then used to estimate the weight associated to each patient for each region/network/network-pair, as follows: $W_{p,r}=\;\frac{\mu_{r}}{S_{p,r},\;}$ where P is the total number of patients which contributed to this specific region/network/network-pair r. For example, if a region/network/network-pair was composed of a total of 200 measurements from 5 patients, an equal distribution would lead to 40 measurements for each patient. We would then need to weight up or down patients having respectively contributed to less or more than 40 measurements. If a patient featured 10 events measured by 2 channels creating a total of 20 measurements, then a weight of 2 should be considered, doubling the importance of each measurement, in order to match the average 40 measures of the group. If for another patient, we selected 20 events and 4 channels, resulting in a total of 80 measurements, a weight of 0.5 should be considered, making each measurement half as important, again to match the average number of measures. Due to the difference in number of phasic/tonic events and wakefulness events we had to compute two sets of weights, one for REM data $W_{p,r}^{REM}$ and one for wakefulness $W_{p,r}^{Wake}$ data. For each measured metric M (i.e. Power or PLV) and each region/network/network-pair r and each segment s, in order to test the differences between phasic and tonic REM segments, we computed the weighted difference between phasic and tonic REM matched segments for each measure $M_{p,r}$ with the corresponding weight $W_{p,r}$ as follows: $D_{p,r,}^{Phasic-Tonic}=\;W_{p,r}^{REM}\left(M_{p,r}^{Phasic}-\;M_{p,r}^{Tonic}\right)$. Such a metric $D_{p,r,}^{Phasic-Tonic}$ was estimated for all available patients, segments, and channels and the resulting $S_{p,r}\times P$ values were pulled together to apply a one sample *t*-test on the weighted values, this is equivalent to a paired weighted *t*-test. On the other hand, since segments were not matched when comparing to wakefulness, a regular unpaired *t*-test was performed to compare distributions of the weighted phasic REM, tonic REM and wakefulness values given by $W_{p,r}^{REM}M_{p,r}^{Phasic},\;W_{p,r}^{REM}M_{p,r}^{Tonic},\;W_{p,r}^{Wake}M_{p,r}^{Wake\;}$ for each available segment of wakefulness, each patient and each channel all pulled together. All p-values were corrected using the false discovery rate (FDR) correction, considering corrected p < 0.05 as significant. The effect size between phasic and tonic REM was assessed using a weighted Cohen’s d $\frac{X}{S}$ where X is the mean of $D_{p,r,}^{Phasic-Tonic}$ over all patients, segment and channels, and S is the corresponding standard deviation of these values. On the other hand, the effect size between phasic/tonic REM and wakefulness segments was given by $\frac{X_{REM}-X_{Wake}}{S_{REM|Wake}}$ where $X_{REM}$ denotes the mean of $W_{p,r}^{REM}M_{p,r}^{Phasic}$ or$,\;W_{p,r}^{REM}M_{p,r}^{Tonic}$ over all patients, segments and channels and $X_{Wake}$ is the mean of $W_{p,r}^{Wake}M_{p,r}^{Wake\;}$ over all patients, segments and channels. $S_{REM|Wake}$ was then computed as the pooled standard deviation of both distributions. Spectral power was assessed for each available region of the 38 MICCAI atlas by using all the channels in the region regardless of laterality. Connectivity analysis was performed both within each network of the Yeo 7 network and between each pair of networks. In addition, we also created a null distribution of the PLV values, by shuffling the network labels of every measure using bootstrap resampling with replacement, 10,000 times. We used these PLV null distributions of connections within a network and between network-pairs and compared them to the actual PLV values we measured within a network and between network-pairs.

## Results

### Segments of phasic and tonic REM sleep

**Figure 3.**
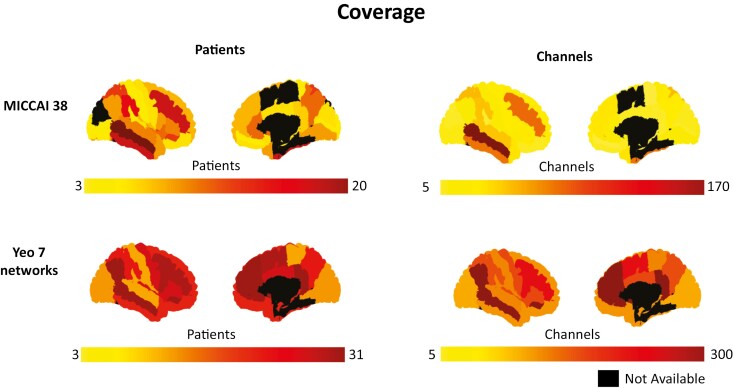
Coverage of regions and networks. Reported are the numbers of channels and patients for each region of the MICCAI 38-region atlas, and YEO 7-network atlas. Regions and networks with less than 3 patients and/or 5 channels were not analyzed and are marked in black as not available.

We report results from 1626 matching tonic and phasic REM events gathered from 31 patients (52.45 ± 34.12 events per patient), with an average duration of 9.12 ± 7.17 seconds. The spatial coverage, including regions and networks with data from at least 3 patients and 5 channels, resulted in reporting from 34 regions and 7 networks (Figure 3).

### REM microstructure displays a spectral power gradient from low to high

**Figure 4.**
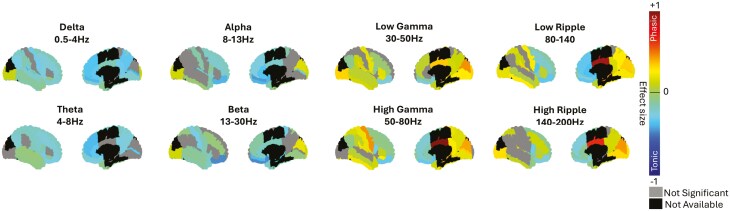
Brain wide power differences between phasic and tonic REM. Effect sizes (Cohen’s *d*) are plotted as colors on each available region exhibiting significant differences between matching time periods of phasic and tonic REM. The effect sizes of significant differences are presented for each power band tested. Significance was set to 0.05 after FDR correction.

We observed significant spectral power differences between phasic and tonic REM across all available regions (p < 0.05, *d* = 0.17 ± 0.20), depending on the frequency bands (Figure 4). Tonic REM exhibited widespread higher power in the low frequency delta and theta bands (p < 0.01, *d* = 0.18 ± 0.08), and high and low delta were similar (Supplementary Figure 1). In contrast, phasic REM showed widespread higher power in the gamma and ripple bands (p < 0.01, *d* = 0.18 ± 0.19), with very high ripples showing similar trends (Supplementary Figure 2) with more regions displaying stronger activation in phasic REM. The alpha and beta bands displayed mixed trends of tonic and phasic strengths, while the Iota band was more similar to low gamma (Supplementary Figure 2). Although there was a general increase in power across both low and high frequencies, not all regions followed this pattern. In the delta and theta ranges, the inferior occipital gyrus and occipital pole exhibited higher power during phasic REM, although with a low effect size (p < 0.05, *d* = 0.07). Conversely, in the gamma and ripple ranges, medial and basal frontal regions showed higher power during tonic REM (e.g. angular gyrus, anterior cingulate, anterior insula, frontal operculum, gyrus rectus and orbital gyri medial frontal cortex, medial segment of superior frontal gyrus, middle frontal gyrus, opercular part of inferior frontal gyrus, superior frontal gyrus and frontal pole temporal pole and planum polare; p < 0.05, *d* = 0.15 ± 0.07).

**Figure 5.**
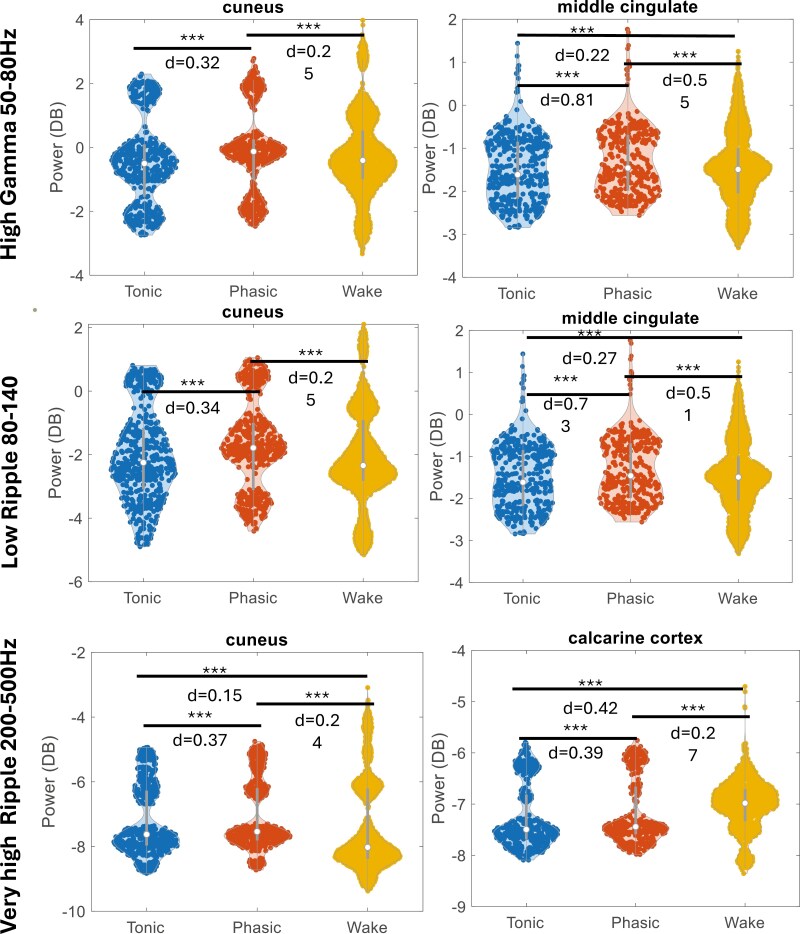
Regional power analysis of high frequencies. The power distributions of every segment for tonic REM, phasic REM, and wakefulness at high frequencies are presented. Regions exhibiting a significant difference and a moderate effect size (*d* > 0.3) between wakefulness and phasic REM are shown. Note that the differences between tonic and phasic REM are matched paired and tests using a paired *t*-test design, while the differences between phasic REM and wakefulness, and tonic REM and wakefulness are based on the distributions and tests using a regular unpaired *t*-test. Results are FDR corrected. Significant results are considered for p < 0.05.

When we examined the bands, we noticed that most of the moderate-stronger effects were at high frequencies (Figure 5) with regions like the cuneus and the middle cingulate displaying a large effect size (*d* > 0.3).

### Diverging local power trends among wakefulness, phasic REM, and tonic REM

**Figure 6.**
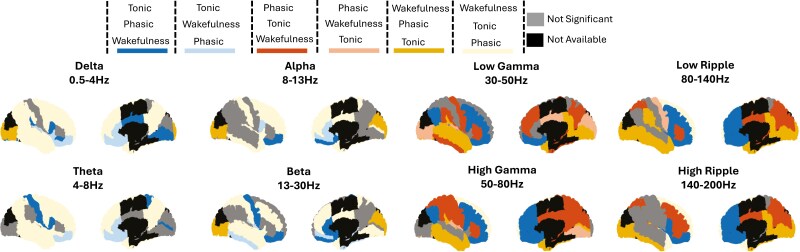
Power trend differences between tonic REM, phasic REM, and wakefulness. The trends for significant differences between wakefulness and phasic and tonic REM have been classified along 6 possible behaviors and color coded for each power band. Every significant regional trend is represented with a color depending on which time period exhibited the highest and lowest power. For example, red represents regions where phasic REM had the highest power, followed by tonic REM, and then wakefulness (phasic > tonic > wakefulness).

Interestingly when examining the differences between phasic and tonic REM when compared to wakefulness, we found that wakefulness was not always higher than REM, but rather behaved differently depending on the region. We aimed to further explore how wakefulness compares to REM microstructure at a local level. To do this, we tested all regions for differences in all frequency bands between wakefulness, tonic REM, and phasic REM. We classified each trend into six categories (tonic > phasic > wakefulness; tonic > wakefulness > phasic; wakefulness > tonic > phasic; wakefulness > phasic > tonic; phasic > tonic > wakefulness; and phasic > wakefulness > tonic, only when all the differences were significant p < 0.05). Our findings revealed regional heterogeneity in these trends (Figure 6). In general, lower frequencies in the delta-theta range demonstrated mixed trends, such as wakefulness > tonic > phasic and tonic > phasic > wakefulness. In contrast, higher frequencies in the gamma and ripple range exhibited trends like wakefulness > phasic > tonic and phasic > tonic > wakefulness. These results suggest that, overall, wakefulness tends to show either higher or lower power compared to both phasic and tonic REM (Supplementary Table S3). However, some regions displayed diverging trends where wakefulness fell between phasic and tonic REM, though mainly in lower frequencies (Table 1)

### Network connectivity is higher during tonic REM compared to phasic REM

**Figure 7.**
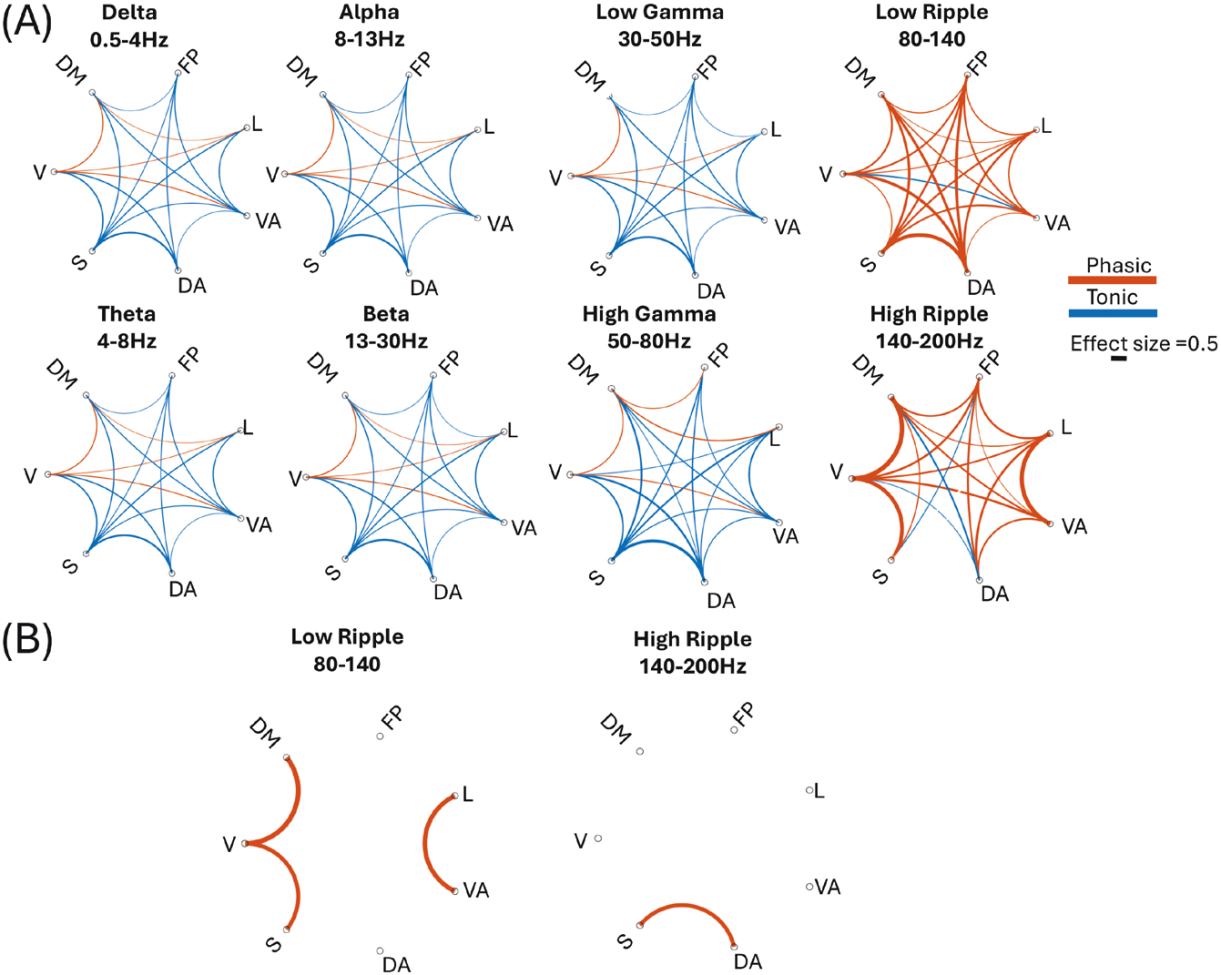
Network-based connectivity differences between phasic and tonic REM. Effect sizes of connectivity, which was significantly different between phasic and tonic REM are plotted as the size and color of connecting lines between each network-pair. Connectivity differences are visualized with blue indicating significantly higher connectivity during tonic REM, and red highlighting regions where connectivity was stronger during phasic REM. (A) all significant results including both low and moderate effect sizes. (B) Only connections which had significant difference with an effect size *d* > 0.3. Significance was set to 0.05 after false discovery rate correction. V—Visual, S—Somatomotor, DA—Dorsal attention, VA—Ventral attention, L—Limbic, FP—Frontoparietal, DM—Default mode network.

**Figure 8.**
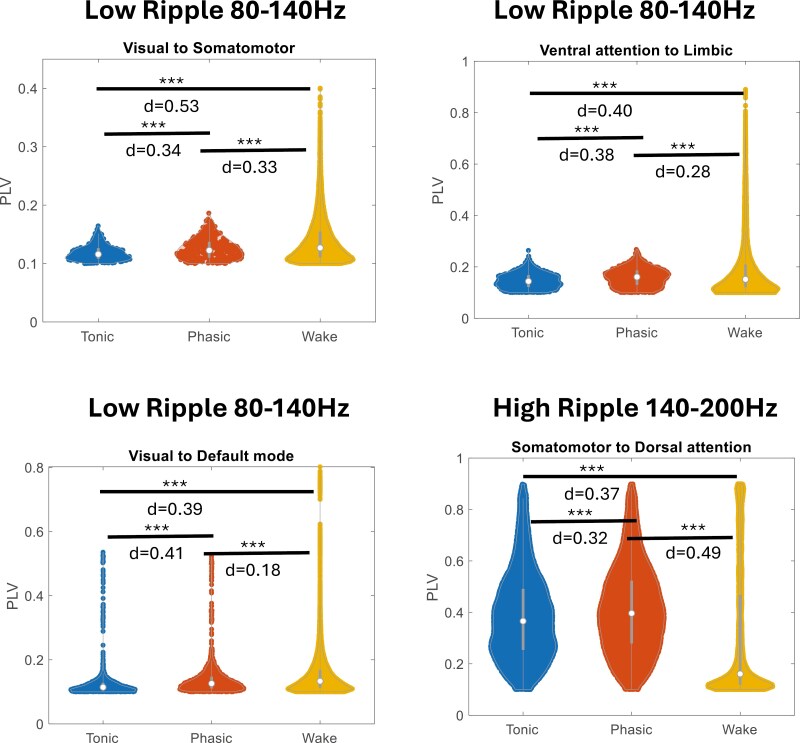
Network analysis of high frequencies. The PLV distributions for wakefulness, phasic REM, and tonic REM sleep at high frequencies are presented. In this figure, we are representing and comparing with the wakefulness state, the distribution of PLV values for the network pairs that exhibited a significant differences between phasic and tonic and with a moderate effect size (*d* > 0.3). Note that the differences between tonic and phasic REM are matched pairs, while the differences between phasic REM and wakefulness, and tonic REM and wakefulness, are based on the distributions.

When investigating between-network connectivity, we observed a low-effect mixed trend of connectivity differences between tonic and phasic REM in all bands (p < 0.01, *d* = 0.08 ± 0.09; Figure 7A). These differences indicated mostly stronger connections during tonic compared to phasic REM in bands ranging from delta to low-ripple frequencies. However, the lower and higher delta bands did not differ significantly (Supplementary Figure 4), and connectivity in the Iota band, located between beta and gamma, similarly showed no significant differences, except for the connectivity between the default mode and limbic networks, which was no longer significant (Supplementary Figure 5).Conversely, connectivity in the low and high ripple bands predominantly demonstrated higher connectivity during phasic REM across most networks. Yet, this trend reversed in the very high ripple rate (Supplementary Figure 5). Additionally, certain network connections, such as visual-ventral attention, visual-default, visual-limbic, and default-limbic networks, exhibited slightly stronger connectivity during phasic REM. However, these findings generally displayed low effect sizes. Focusing specifically on moderate effect sizes (*d* > 0.3), we noted acute increases predominantly in higher frequency bands (Figure 7B). Specifically, stronger connectivity during phasic REM was observed in the low ripple band between the visual and default mode networks, visual and somatomotor networks, and limbic and ventral attention networks. Furthermore, the high ripple band consistently showed higher connectivity during phasic REM across all networks (Figure 8).

When examining within network connectivity we found similar trends in within-network connectivity, mirroring the patterns observed in between-network connectivity. Specifically, tonic REM showed generally stronger connections than phasic REM in bands from delta to low-ripple frequencies, though with low effect sizes (p < 0.01, *d* = 0.08 ± 0.09; Figure 7A). In contrast, connectivity within networks in the low and high ripple bands also exhibited higher connectivity during phasic REM, consistent with the between-network findings. Again, this trend reversed at the very high ripple rate (Supplementary Figure 5). When considering moderate effect sizes (*d* > 0.3), within-network connectivity displayed acute increases exclusively at higher frequencies (Figure 7B). High ripple bands, specifically, demonstrated consistently higher connectivity during phasic REM within all networks (Figure 8).

When considering the trend in relations to wakefulness we observed that overall connectivity during wakefulness was higher during REM in 72% of networks connections and did not vary between bands. We observed no instances where networks have displayed wakefulness as in between tonic and phasic REM.

### Early sleep cycles compared to late sleep cycles

We performed a sub analysis of the early part of the night (cycles 1-2) compared to the late part of the night (last 2 cycles). We found that for our spectral analysis across bands and regions the same regions displayed differences between the phasic and tonic REM, with similar effect size when comparing the power in phasic versus tonic REM (early: *d* = 0.15 ± 0.15, late: *d* = 0.18 ± 0.18). In addition, the same trend of tonic predominance in lower frequencies (early: *d* = 0.14 ± 0.16, late: *d* = 0.15 ± 0.13), and phasic in higher frequencies (early: *d* = 0.16 ± 0.07, late: *d* = 0.20 ± 0.10) were seen in both early and late parts of the night. However, differences between tonic and phasic REM tended to have slightly larger effect sizes during late sleep. We also observed that connectivity differences between phasic and tonic REM were similar between early and late sleep in all networks and frequencies (early: 0.10=±0.13, late: *d* = 0.10 ± 0.12). In addition, the same trends, of stronger connectivity during tonic REM in the lower to gamma band (early: *d* = 0.08 ± 0.07, late: *d* = 0.06 ± 0.05), and stronger connectivity during phasic in higher frequencies (early: *d* = 0.21 ± 0.19, late: *d* = 0.17 ± 0.20) were seen in both early and late parts of the night, but tended to have slightly lower effect sizes during late sleep.

## Discussion

In recent years investigation into REM microstructure has started to gain interest [8]. Thus far, most evidence concerning the difference between phasic and tonic REM came from scalp EEG or intracranial EEG in a limited number of regions. Here we are reporting the results of the first comprehensive spatial investigation of REM microstructure using simultaneous scalp and intracranial EEG recordings. Investigating 34 regions and 7 networks of the brain to identify differences between tonic and phasic REM, and comparing them to wakefulness, we found that (1) there is a spectral gradient between tonic and phasic REM alongside regional differences which depend on the frequency band, (2) the power during wakefulness displayed regional variability in relation to tonic and phasic REM (3) connectivity is generally stronger during tonic REM compared to phasic REM, with opposing trends mainly for the visual network with stronger connectivity during phasic periods.

### Widespread and region-specific spectral differences between phasic and tonic REM

We found a robust widespread spectral gradient from tonic to phasic REM, yet some regions diverged from the general trend (Figures 4-5). Our findings are aligned with the concept that sleep is both a global and local phenomenon [27, 33, 36]. We found that REM microstructure exhibits both global differences—manifested by a general trend of stronger power in lower frequencies during tonic REM and stronger power in higher frequencies during phasic REM (Figures 4 and 5)—suggestive of a global phenomenon. At the same time, our analysis displays regional differences compared to the global trend, such as occipito-temporal regions showing higher power during phasic REM at low frequencies and medial-basal frontal regions showing higher power during tonic REM at high frequencies. These findings are consistent with previous scalp EEG studies, which suggested the presence of a power gradient from low to high frequencies between tonic and phasic REM sleep [8, 20, 21, 46–48]. Interestingly, we found an increase in high-frequency activity in phasic REM sleep in widespread regions, including the primary sensorimotor cortex and in parieto-occipital areas and well as in the dorsolateral prefrontal cortex. The activation of the somatomotor cortex during phasic REM sleep, contrasting with muscle atonia associated inhibition of muscle activity by the brainstem during this state, has been highlighted in the sEEG study of De Carli et al. Frequencies above 32 Hz, however were not analyzed in this work [31]. These finding are consistent with the observation that motor-behavioral episodes more likely occur during phasic versus tonic REM sleep in the context of REM sleep behavior disorder [49] although somatomotor cortical network activation might be even enhanced in this context [50]. The increase in gamma activity in both primary and associative cortices during phasic REM sleep echoes several studies which investigated brain activation locked on rapid eye movements; For instance, a magnetoencephalography study reported increased activity in the gamma band in areas involved in visuomotor processing following rapid eye movements [51]. Functional magnetic resonance imaging and positron emission tomography studies reported widespread activation in visual and non-visual sensory cortices, motor cortex and several associative areas including the cingulate and retrosplenial cortex and deactivation of the default mode network during phasic REM [52, 53]. The parieto-occipital cortex has been indeed identified as a “hot zone” associated with dream recall; the observation of high gamma activity in this region may be related to the reported increase in intensity, vividness and visual imaging of dreams reported after awakening in phasic versus tonic REM sleep [54–57] or correlated with rapid eye movements. The current study lacks a comprehensive scalp EEG coverage, thus limiting our ability to address in full previous scalp findings [46] and how they relate to activity in sEEG. To rigorously evaluate this, a complete brain coverage utilizing at least a standard 10-20 EEG montage would be necessary.

### Comparison of REM microstructure with wakefulness at the global and local scale

We observed that wakefulness varied in its relative power according to the considered frequency bands when compared to phasic and tonic REM (Figure 6). This is in line with the observation that REM sleep and wakefulness share similar electrographic signatures, characterized by higher-frequency content and lower amplitude voltage [58]. When comparing REM sleep and wakefulness using sEEG in the neocortex, previous studies showed that wakefulness had higher power in the gamma band but slightly lower power than REM in the beta band [29]. Given the dependence on frequency band and location, we analyzed our data with respect to wakefulness. We found that, in some cases, tonic REM acts as a middle point between phasic REM and wakefulness, while in other regions, wakefulness itself falls between tonic and phasic REM (Table 1). Generally, in the lower-frequency bands, frontal and temporal regions tended to show either higher wakefulness power than the entirety of REM or lower power, with medial frontal regions placing wakefulness between phasic and tonic REM. Similarly, in the higher frequencies, the same trend was observed, though with the order between phasic and tonic REM inverted. Notably, a few medial areas showed wakefulness positioned between tonic and phasic REM as well. This resemblance in high-frequency activity between wakefulness and phasic REM sleep echoes results of prior studies showing increased neuronal firing in the medial temporal lobe in REM sleep after rapid eye movements, which likely mimics visual input processing [59]. In addition, an increased high frequency activity in the motor cortex during phasic REM, that resembled the activity present during active motor movements was shown [31]. Future studies can build upon the detailed findings presented here, correlating these granular differences with behavioral outcomes observed in REM sleep related research.

**Table 1.** List of regions where wakefulness displayed an intermediate power between tonic and phasic REM. The regions are listed for each band in which wakefulness showed a power level between phasic and tonic REM. Only regions displaying a significant difference (p < 0.05) across all comparisons are included. In other words, wakefulness differs from both phasic and tonic REM, and tonic REM differs from phasic REM.

Band	Regions
*Low Delta* *(0.5-2Hz)*	anterior insula, medial frontal cortex, posterior insula, precuneus, transverse temporal gyrus
*High Delta* *(2-4Hz)*	anterior cingulate, medial frontal cortex, middle frontal gyrus, supramarginal gyrus, transverse temporal gyrus, triangular part of inferior frontal gyrus
*Delta* *(0.5-4Hz)*	anterior cingulate, medial frontal cortex, middle frontal gyrus, supramarginal gyrus, transverse temporal gyrus, triangular part of inferior frontal gyrus
*Theta* *(4-8Hz)*	anterior cingulate, gyrus rectus and orbital gyri, medial frontal cortex
*Alpha* *(8-13Hz)*	anterior cingulate, frontal operculum, medial frontal cortex, opercular part of inferior frontal gyrus
*Beta* *(13-30Hz)*	central operculum, hippocampus, inferior temporal gyrus, medial frontal cortex
*Iota* *(25-35Hz)*	cuneus, lingual gyrus and occipital fusiform gyrus, posterior insula, superior frontal gyrus and frontal pole
*Low Gamma* *(30-50Hz)*	cuneus, inferior occipital gyrus and occipital pole, middle cingulate, planum temporale, posterior insula
*High Gamma* *(50-80Hz)*	central operculum, lingual gyrus and occipital fusiform gyrus, planum temporale, posterior insula
*Low Ripple* *(80-140Hz)*	precentral gyrus
*High Ripple* *(140-200Hz)*	postcentral gyrus
*Very High Ripple* *(200-500Hz)*	precuneus, superior parietal lobule

### Functional connectivity differences between tonic and phasic REM

When we examined the functional connectivity patterns using PLV, between the Yeo7 networks [38] in different bands, we found a consistent pattern of tonic connectivity being higher than phasic between and within most network connections and most frequency bands, except for the high ripple band. This confirms previous findings from studies in the field, keeping in mind that previous investigations were based on scalp EEG and did not explore gamma activity above > 50Hz [20, 21]. Connectivity results also showed a widespread trend of stronger connectivity in tonic REM in all bands except the high ripple band (Figures 6 and 7), while demonstrating local variability, such as for connections involving the visual and default mode networks exhibiting higher connectivity during phasic REM. The observed increase in connectivity between the somatomotor network and other networks during phasic REM sleep (Figure 7B) highlights a promising avenue for future research on diseased REM sleep, such as present in REM behavior disorder. Specifically, examining the connectivity within and towards this network during phasic REM in patients may reveal significant differences, given that connectivity alterations have previously been implicated in this disorder [60]. However, we were able to identify an increased connectivity between and within networks related to the visual network and, in some bands, to the dorsal or ventral attention networks. Although literature on this topic is limited, studies on thalamocortical functional connectivity have also shown higher connectivity during phasic REM periods [32], as well as during sawtooth waves, which mostly overlap with bursts of rapid eye movements [61]. Subcortical structures were not available for analysis in the current study. Previous research has explored the role of subcortical regions, such as the anterior thalamic nucleus [32] showing an increased thalamocortical connectivity during tonic REM. Future studies are necessary to determine whether other thalamic nuclei, such as the ventral and posterior nuclei, also exhibit differences between these REM states. When using simultaneous EEG and functional magnetic resonance imaging recordings, it was also shown that the activity in thalamocortical networks increased during phasic REM in seven subjects with a sleep deprivation protocol [12]. Unfortunately, we were unable to confirm this observation as thalamic recordings were not available in our study. However, it is possible that functional connectivity between the visual network’s with other cortical networks may parallel thalamic connectivity with cortical networks, as both systems exert some control over visual processing [62]. Interestingly, the high ripple band showed a widespread increase of functional connectivity in all networks during phasic REM. Such frequency-, network- and substate-related heterogeneity may explain the recently reported complex patterns of functional connectivity during REM sleep [63]. In a recent study [64] examining thalamocortical and intracortical network changes between periods of delta activity in the thalamus and periods of rapid activity, it was found that cortical connectivity was lower during delta thalamic REM activity compared to REM with rapid thalamic activity, as well as compared to wakefulness, primarily during tonic REM.

### High frequency bands display opposing trends compared to lower bands

We noticed that for both spectral and functional connectivity results, the difference between tonic and phasic REM was particularly significant in high frequencies. This observation is interesting in relation to the recent increase in interest in their role in sleep research. High frequencies were attributed importance for their role in cognitive functions such as memory [65, 66], attention [67], language [68] and consciousness [69, 70]. When the upper range was explored in the context of REM microstructure, gamma band activity was identified as an important differentiator between tonic and phasic REM [20]. When examined using sEEG, further trends began to emerge [31]. Our analysis of high frequencies revealed opposing trends that changed between tonic and phasic REM, highlighting the importance of investigating these higher frequency ranges, which are only accessible through sEEG. We found that high frequencies exhibited greater power during phasic REM, particularly in the very high-frequency bands (Figures 4–6). In addition, we found that the ripple band showed a higher functional connectivity in phasic compared to tonic REM between and within all networks (Figures 6 and 7). The importance of these higher bands has been further emphasized by recent studies of transient high-frequency oscillations above 80 Hz, which demonstrated distinct patterns between sleep and wakefulness [71, 72], as well as broad-band differences during transitions from REM to wakefulness [39] The specific changes in connectivity with tonic REM dominated by lower frequencies and phasic REM by high frequencies might intersect with the previous finding and point to difference in the state of consciousness between tonic and phasic REM. Additionally, our group recently explored the high frequency band (80–200 Hz) in REM sleep in the context of sawtooth waves, revealing spatial heterogeneity of REM sleep. Sawtooth waves were found to correlate with a strong and widespread increase in the high frequency oscillations > 80Hz band [73], alongside increased thalamocortical connectivity [61]. It has been showed that desynchronized EEG, as particularly present during phasic REM as the most desynchronized state compared to tonic REM sleep, might be protective against interictal activity and seizures [74, 75].

### Limitations

Due to the spatial sparsity of SEEG recordings, we were unable to survey all areas of the brain, particularly subcortical regions, which were not available to us. Additionally, we cannot entirely rule out the effect of epilepsy on sleep, given that multi-electrode SEEG is only available in the context of epilepsy presurgical evaluation. However, we believe that this influence would be heterogeneous given the different localizations of the epileptogenic zones and likely make it harder to find significant changes. Also, we carefully selected only channels that showed no or very scarce epileptic activity, as verified by a board-certified neurophysiologist as well as an automatic spike detector. Importantly, phasic and tonic substates may not encompass all the heterogeneity of REM sleep; this state also differs in the presence/absence of sawtooth waves [35, 61], as well as in the pattern of thalamic activity, which does not overlap with traditional phasic/tonic states [64]. We did not have access to a sufficient number of channels per hemisphere to thoroughly investigate hemispheric differences between regions. Future studies are needed to better understand these hemisphere-dependent differences. To do so, multicenter datasets are required to achieve the needed coverage. This study relies exclusively on sEEG recordings as well as a limited scalp EEG sampling for sleep scoring; therefore, examining whole-cortex coverage was not feasible. Future studies utilizing full scalp EEG coverage will be necessary to comprehensively address this issue.

## Conclusions

REM is not a singular state; tonic and phasic REM differ in both their spectral and functional connectivity patterns in a frequency- and region-dependent manner. A spectral gradient from tonic to phasic REM is evident in the signal power, with slower frequencies predominating tonic REM and faster frequencies predominating phasic REM. In contrast, the functional connectivity patterns remain similar across most frequency bands, showing higher connectivity in tonic as opposed to phasic REM sleep.

## Supplementary material

Supplementary material is available at *SLEEP* online.

zsaf133_suppl_Supplementary_Figures_1-5

## Data Availability

Processed data will be available upon request to the corresponding author of this work. The raw SEEG data are not available for open datasharing, as open datasharing was not part of the IRB approval for this project.
