# Supplementary material for: Spectral and network investigation reveals distinct power and connectivity patterns between phasic and tonic REM sleep
Source: Sleep. 2025 May 21;48(8):zsaf133. doi: 10.1093/sleep/zsaf133 (PMC12351276; doi:10.1093/sleep/zsaf133)
Supplement: zsaf133_suppl_Supplementary_Figures_1-5 [file zsaf133_suppl_supplementary_figures_1-5.docx]

Supporting Information: Spectral and network investigation reveals distinct power and connectivity patterns between phasic and tonic REM sleep

Tamir Avigdor^1,2^, Laure Peter-Derex^3^, Alyssa Ho^4^, Katharina Schiller^1^, Yingqi Wang^1^, Chifaou Abdallah^1,2^, Edouard Delaire^5^, Kassem Jaber^4,6^, Vojtech Travnicek^7,8^, Christophe Grova^2,5^, Birgit Frauscher^1,4,6^

1. Analytical Neurophysiology Lab, McGill University, Montreal, Quebec, Canada.

2. Multimodal Functional Imaging Lab, Biomedical Engineering Department, McGill University, Montreal, Quebec, Canada.

3. Center for Sleep Medicine, Croix-Rousse Hospital, Hospices Civils de Lyon, Lyon, France; Lyon Neuroscience Research Center, PAM Team, INSERM U1028 / CNRS UMR 5292 / Lyon 1 University, Lyon, France.

4. Analytical Neurophysiological Lab, Department of Neurology, Duke University, Durham, North Carolina, USA.

5. Multimodal Functional Imaging Lab, Department of Physics, PERFORM Center / School of Health, Concordia University, Montreal, Quebec, Canada.

6. Department of Biomedical Engineering. Duke Pratt School of Engineering, Durham, North Carolina, USA.

7. Institute of Scientific Instruments, Czech Academy of Sciences, Brno, Czech Republic.

8. International Clinical Research Center, St Anne's University Hospital, Brno, Czech Republic.

Corresponding author**:**

Birgit Frauscher, MD, PhD
Professor of Neurology

Director, Duke Comprehensive Epilepsy Center
Duke University Medical Center

Email: birgit.frauscher@duke.edu

Tel: 919 613-9386


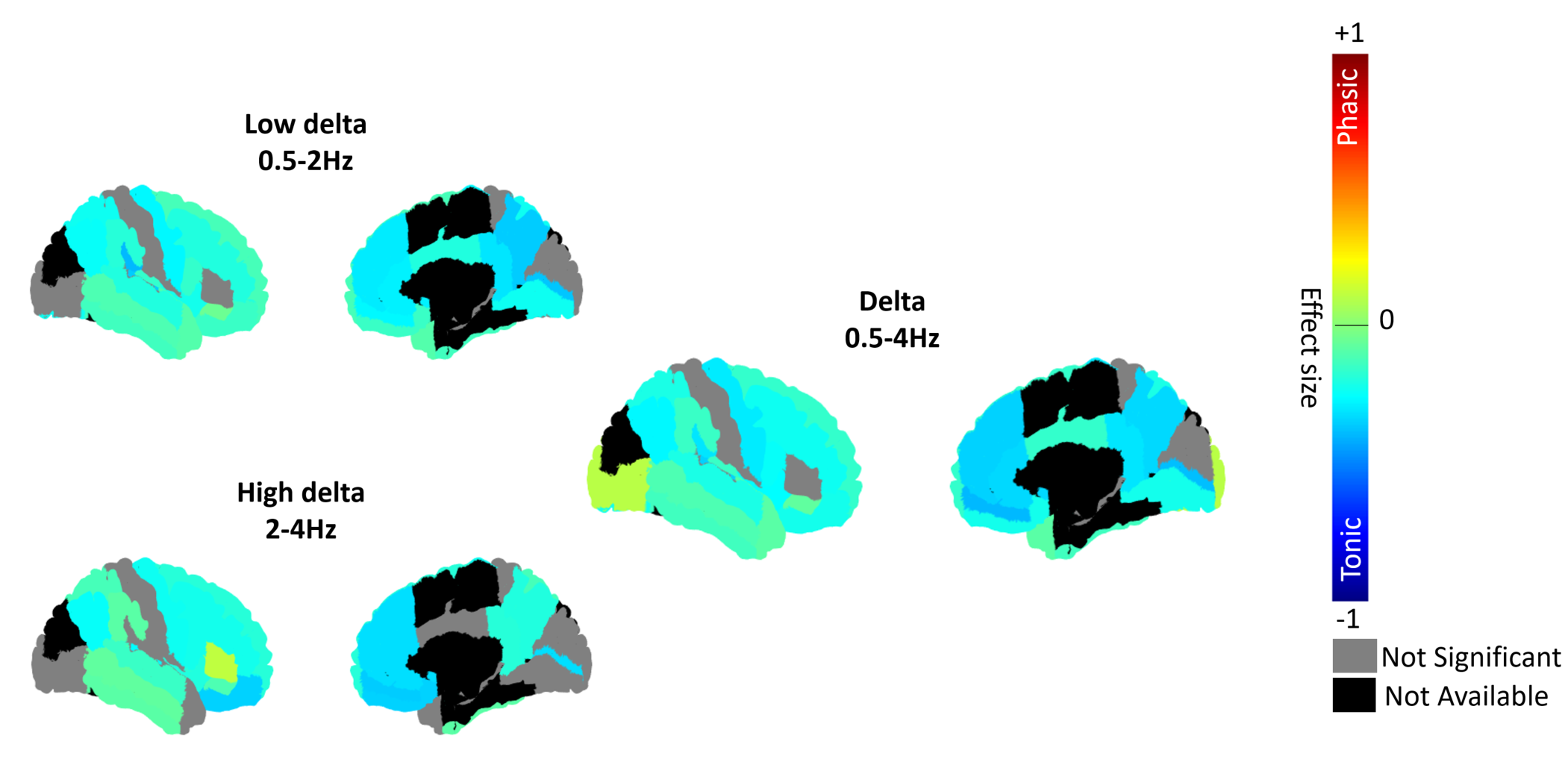


**Figure 1. Differences in delta band power between phasic and tonic REM.** Effect sizes (Cohen's d) are plotted as colors on each available region exhibiting significant differences between matching time periods of phasic and tonic REM. The effect size of regions with significant differences are presented for each power band tested. Significance was set to 0.05 after FDR correction. Note: low delta (p < 0.01, d = 0.25 ± 0.11) and high delta (p < 0.01, d = 0.20 ± 0.15) were similar, only differing in the following regions: central operculum, lingual gyrus and middle cingulate, occipital fusiform gyrus, orbital part of inferior frontal gyrus, superior frontal gyrus and frontal pole, triangular part of inferior frontal gyrus.


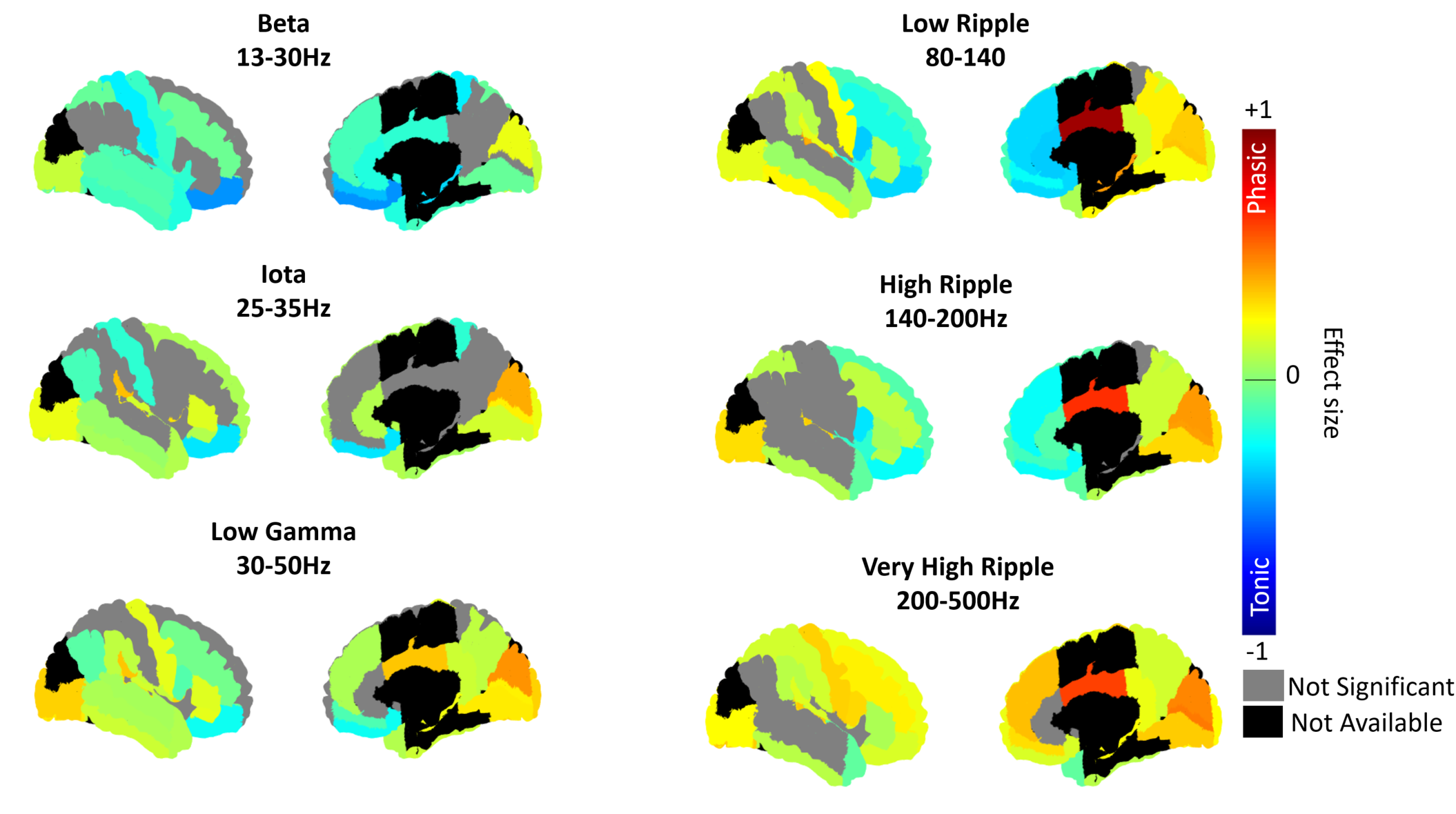


**Figure 2. Differences in high frequency band power between phasic and tonic REM.** Depicted is the Iota band next to the beta and low gamma band, as well as the very high ripple band compared to the other ripple bands. Effect sizes (Cohen's d) are plotted as colors on each available region with significant differences between matching time periods of phasic and tonic REM. The significant differences are presented for each power band tested. Significance was set to 0.05 after FDR correction.

| Region | Patients | Total channel count | Channels per patients | Total Phasic/Tonic segments | Phasic/Tonic segments  per patient |
| --- | --- | --- | --- | --- | --- |
| superior and middle occipital gyri | 1 | 9 | 9 | 58 | 58 |
| inferior occipital gyrus and occipital pole | 3 | 12 | 4 , 4 , 4 | 170 | 103, 49, 18 |
| cuneus | 4 | 11 | 2, 1, 5, 3 | 219 | 89, 58, 54, 18 |
| calcarine cortex | 3 | 9 | 3, 1, 5 | 161 | 89, 54, 18 |
| lingual gyrus and occipital fusiform gyrus | 7 | 23 | 3, 4, 4, 4, 3, 2, 3 | 458 | 57, 88, 103, 54, 49, 18, 89 |
| postcentral gyrus (including medial segment) | 3 | 15 | 1, 11, 3 | 130 | 21, 20, 89 |
| superior parietal lobule | 11 | 49 | 2, 5, 3, 6, 1, 2, 7, 1, 11, 10, 1 | 550 | 61, 89, 75, 34, 12, 58, 54, 49, 9, 20, 89 |
| parietal operculum | 3 | 7 | 1, 3, 3 | 136 | 88, 28, 20 |
| supramarginal gyrus | 14 | 46 | 1, 1, 3, 3, 2, 3, 4, 1, 3, 2, 10, 8, 4, 1 | 666 | 61, 89, 68, 75, 88, 34, 57, 12, 28, 22, 54, 49, 9, 20 |
| angular gyrus | 7 | 22 | 2, 4, 5, 1, 7, 1, 2 | 291 | 39, 68, 34, 58, 54, 18, 20 |
| precuneus | 9 | 29 | 3, 2, 2, 3, 3, 7, 1, 3, 5 | 445 | 89, 75, 88, 34, 58, 54, 9, 18, 20 |
| posterior cingulate | 5 | 9 | 2, 1, 2, 1, 3 | 272 | 89, 88, 34, 12, 49 |
| anterior insula | 9 | 16 | 4, 2, 1, 1, 2, 1, 1, 3, 1 | 518 | 75, 35, 28, 103, 54, 49, 65, 20, 89 |
| posterior insula | 4 | 25 | 4, 6, 10, 5 | 124 | 28, 22, 54, 20 |
| gyrus rectus and orbital gyri | 4 | 10 | 3, 1, 2, 4 | 203 | 35, 54, 49, 65 |
| anterior cingulate | 9 | 24 | 2, 2, 4, 2, 3, 3, 3, 4, 1 | 472 | 61, 28, 39, 57, 12, 67, 54, 65, 89 |
| middle cingulate | 4 | 10 | 2, 5, 1, 2 | 158 | 29, 28, 12, 89 |
| supplementary motor cortex | 2 | 2 | 1, 1 | 82 | 28, 54 |
| medial frontal cortex | 3 | 6 | 1, 3, 2 | 207 | 39, 103, 65 |
| central operculum | 6 | 14 | 1, 1, 4, 1, 2, 5 | 301 | 75, 28, 21, 103, 54, 20 |
| frontal operculum | 11 | 25 | 1, 2, 1, 4, 2, 3, 1, 3, 1, 4, 3 | 532 | 29, 75, 35, 28, 67, 21, 54, 49, 65, 20, 89 |
| opercular part of inferior frontal gyrus | 7 | 26 | 2, 2, 3, 10, 5, 2, 2 | 327 | 29, 75, 28, 21, 65, 20, 89 |
| triangular part of inferior frontal gyrus | 8 | 31 | 4, 2, 1, 4, 1, 10, 6, 3 | 592 | 179, 75, 63, 35, 67, 21, 103, 49 |
| orbital part of inferior frontal gyrus | 11 | 30 | 1, 1, 3, 3, 7, 2, 3, 2, 3, 2, 3 | 728 | 61, 179, 39, 63, 57, 35, 67, 21, 103, 54, 49 |
| middle frontal gyrus | 16 | 76 | 2, 1, 5, 1, 2, 5, 2, 5, 2, 7, 12, 4, 8, 5, 4, 11 | 927 | 61, 179, 29, 75, 28, 88, 39, 35, 28, 67, 21, 54, 49, 65, 20, 89 |
| superior frontal gyrus and frontal pole | 6 | 27 | 3, 7, 6, 2, 1, 8 | 335 | 39, 67, 21, 54, 65, 89 |
| medial segment of superior frontal gyrus | 6 | 32 | 3, 1, 7, 9, 8, 4 | 326 | 39, 12, 67, 54, 65, 89 |
| medial segment of precentral gyrus | 2 | 5 | 2, 3 | 29 | 9, 20 |
| precentral gyrus | 4 | 28 | 5, 7, 15, 1 | 104 | 21, 54, 9, 20 |
| superior temporal gyrus | 8 | 45 | 1, 1, 3, 2, 10, 14, 5, 9 | 354 | 57, 28, 22, 21, 103, 54, 49, 20 |
| middle temporal gyrus | 20 | 166 | 9, 2, 12, 2, 3, 2, 5, 9, 3, 4, 12, 10, 5, 6, 2, 40, 11, 1, 25, 3 | 1001 | 61, 39, 36, 68, 88, 34, 63, 34, 57, 12, 28, 22, 67, 21, 58, 103, 54, 49, 18, 89 |
| inferior temporal gyrus | 16 | 72 | 1, 2, 2, 2, 2, 5, 1, 6, 2, 1, 4, 8, 8, 14, 12, 2 | 855 | 61, 39, 36, 68, 88, 63, 12, 28, 22, 67, 58, 103, 54, 49, 18, 89 |
| temporal pole and planum polare | 7 | 33 | 4, 2, 2, 9, 2, 5, 9 | 344 | 68, 28, 22, 103, 54, 49, 20 |
| transverse temporal gyrus | 3 | 5 | 2, 1, 2 | 150 | 68, 28, 54 |
| planum temporale | 4 | 10 | 2, 1, 2, 5 | 131 | 68, 22, 21, 20 |
| fusiform and parahippocampal gyri | 1 | 0 |  | 0 |  |
| hippocampus | 4 | 5 | 1, 1, 2, 1 | 180 | 34, 22, 21, 103 |
| amygdala | 1 | 0 |  | 0 |  |

**Table 1. Regional coverage.** Listed are all the MICCAI38 atlas regions with the number of patients, channels and phasic/tonic segments for each region, and to how many of them each patient contributed. Note: this proportion was used to weight the test and effect sizes such that each paint has equal contribution regardless of the number of channels and phasic/tonic segments contributed.

| Networks | Patients | Total channel count | Channels per patients | Total Phasic/Tonic segments | Phasic/Tonic segments  per patient |
| --- | --- | --- | --- | --- | --- |
| Default mode | 10 | 734 | 89, 57, 88, 22, 58, 103, 54, 49, 18, 89 | 627 | 15, 15, 6, 1, 21, 28, 171, 36, 435, 6 |
| Visual -Somatomotor | 3 | 142 | 103, 54, 49 | 206 | 32, 65, 45 |
| Visual -Dorsal attention | 9 | 750 | 57, 88, 63, 58, 103, 54, 49, 18, 89 | 579 | 9, 16, 4, 5, 57, 298, 69, 272, 20 |
| Visual -Ventral attention | 6 | 306 | 22, 103, 54, 49, 18, 89 | 335 | 6, 72, 33, 117, 30, 48 |
| Visual -Limbic | 6 | 252 | 22, 103, 54, 49, 18, 89 | 335 | 8, 79, 36, 63, 58, 8 |
| Visual -Frontoparietal | 7 | 513 | 88, 63, 103, 54, 49, 18, 89 | 464 | 4, 3, 97, 246, 107, 48, 8 |
| Visual -Default mode | 7 | 508 | 22, 58, 103, 54, 49, 18, 89 | 393 | 24, 2, 158, 143, 99, 10, 72 |
| Somatomotor | 9 | 1237 | 68, 28, 22, 21, 103, 54, 49, 9, 20 | 374 | 10, 28, 10, 36, 6, 300, 10, 171, 666 |
| Somatomotor -Dorsal attention | 7 | 839 | 57, 28, 21, 54, 49, 9, 20 | 238 | 4, 40, 21, 532, 20, 68, 154 |
| Somatomotor -Ventral attention | 8 | 484 | 57, 22, 21, 103, 54, 49, 9, 20 | 335 | 5, 35, 52, 12, 185, 65, 22, 108 |
| Somatomotor -Limbic | 7 | 265 | 68, 57, 28, 22, 21, 54, 49 | 299 | 15, 8, 32, 20, 57, 98, 35 |
| Somatomotor -Frontoparietal | 8 | 839 | 57, 28, 21, 103, 54, 49, 9, 20 | 341 | 5, 40, 189, 24, 469, 55, 24, 33 |
| Somatomotor -Default mode | 9 | 880 | 68, 57, 28, 22, 21, 103, 54, 49, 20 | 422 | 40, 2, 64, 60, 171, 56, 389, 50, 48 |
| Dorsal attention | 20 | 856 | 61, 36, 89, 57, 75, 88, 34, 63, 57, 12, 28, 21, 58, 103, 54, 49, 9, 18, 20, 89 | 1021 | 3, 1, 21, 1, 3, 10, 21, 6, 6, 1, 10, 3, 21, 45, 351, 45, 45, 136, 91, 36 |
| Dorsal attention -Ventral attention | 13 | 496 | 61, 89, 88, 34, 57, 21, 103, 54, 49, 9, 18, 20, 89 | 692 | 6, 12, 3, 7, 5, 6, 90, 60, 102, 10, 17, 70, 108 |
| Dorsal attention -Limbic | 8 | 254 | 57, 28, 21, 103, 54, 49, 18, 89 | 419 | 8, 20, 15, 89, 34, 42, 28, 18 |
| Dorsal attention -Frontoparietal | 18 | 720 | 61, 36, 89, 75, 88, 34, 63, 57, 28, 21, 58, 103, 54, 49, 9, 18, 20, 89 | 952 | 2, 6, 11, 9, 5, 8, 12, 5, 25, 9, 12, 123, 307, 78, 20, 40, 30, 18 |
| Dorsal attention -Default mode | 12 | 805 | 61, 34, 57, 12, 28, 21, 58, 103, 54, 49, 20, 89 | 586 | 23, 49, 1, 2, 40, 27, 14, 194, 199, 66, 28, 162 |
| Ventral attention -Ventral attention | 2 | 752 | 61, 29, 89, 75, 28, 88, 63, 39, 57, 12, 35, 28, 22, 67, 21, 103, 54, 49, 65, 9, 20, 89 | 1103 | 1, 1, 3, 3, 15, 6, 6, 3, 10, 10, 28, 28, 28, 6, 21, 55, 190, 78, 3, 1, 190, 66 |
| Ventral attention -Limbic | 8 | 246 | 63, 57, 28, 22, 67, 21, 103, 54 | 415 | 16, 15, 32, 20, 12, 27, 36, 88 |
| Ventral attention -Frontoparietal | 18 | 943 | 29, 89, 75, 88, 63, 39, 57, 35, 28, 67, 21, 103, 54, 49, 65, 9, 20, 89 | 980 | 11, 3, 15, 7, 12, 3, 9, 31, 65, 31, 104, 82, 421, 32, 24, 4, 85, 4 |
| Ventral attention -Default mode | 17 | 986 | 29, 75, 28, 88, 63, 12, 35, 28, 22, 67, 21, 103, 54, 49, 65, 20, 89 | 848 | 2, 2, 4, 3, 8, 5, 4, 64, 60, 33, 71, 175, 359, 30, 18, 60, 88 |
| Limbic | 17 | 452 | 39, 36, 68, 63, 57, 12, 35, 28, 22, 67, 21, 103, 54, 49, 65, 18, 89 | 826 | 3, 3, 3, 28, 28, 1, 15, 6, 6, 6, 21, 153, 15, 55, 3, 105, 1 |
| Limbic -Frontoparietal | 16 | 797 | 179, 39, 36, 75, 63, 57, 12, 35, 67, 21, 103, 54, 49, 65, 18, 89 | 962 | 1, 3, 9, 9, 28, 30, 6, 48, 29, 57, 216, 126, 149, 24, 60, 2 |
| Limbic -Default mode | 19 | 904 | 179, 39, 36, 68, 75, 63, 57, 12, 35, 28, 22, 67, 21, 103, 54, 49, 65, 18, 89 | 1080 | 4, 7, 23, 24, 5, 16, 2, 12, 6, 32, 24, 9, 87, 369, 86, 86, 54, 22, 36 |
| Frontoparietal | 26 | 1292 | 61, 179, 36, 29, 89, 75, 28, 88, 34, 63, 39, 57, 12, 35, 28, 67, 21, 58, 103, 54, 49, 65, 9, 18, 20, 89 | 1406 | 10, 21, 3, 21, 1, 36, 1, 10, 1, 6, 15, 10, 3, 28, 45, 120, 351, 15, 78, 300, 153, 28, 1, 6, 28, 1 |
| Frontoparietal -Default mode | 20 | 1193 | 61, 179, 29, 75, 28, 88, 34, 63, 12, 35, 28, 67, 21, 58, 103, 54, 49, 65, 20, 89 | 1158 | 45, 28, 3, 18, 2, 12, 14, 2, 6, 1, 80, 123, 313, 12, 134, 207, 127, 16, 26, 24 |
| Default mode | 26 | 2373 | 61, 179, 39, 36, 68, 75, 28, 88, 34, 63, 34, 39, 57, 12, 28, 22, 67, 21, 58, 103, 54, 49, 65, 18, 20, 89 | 1407 | 55, 6, 28, 36, 66, 10, 1, 3, 78, 1, 45, 10, 10, 15, 28, 66, 153, 253, 1, 820, 300, 55, 153, 21, 6, 153 |

**Table 2. Network coverage.** Listed are all the Yeo7 atlas network and network pairs with the number of patients, channels and phasic/tonic segments for each region, and to how many of them each patient contributed. Note: this proportion was used to weight the test and effect sizes such that each patient has equal contribution regardless of the number of channels and phasic/tonic segments contributed.

| **Band** | **REM> Wakefulness** | **REM< Wakefulness** |
| --- | --- | --- |
| Low Delta | 14 (41%) | 9 (26%) |
| High Delta | 11 (32%) | 11 (32%) |
| Delta | 14 (41%) | 10 (29%) |
| Theta | 14 (41%) | 9 (26%) |
| Alpha | 25 (73%) | 2 (5%) |
| Beta | 19 (55%) | 6 (17%) |
| Iota | 8 (23%) | 9 (26%) |
| Low Gamma | 5 (14%) | 14(41%) |
| High Gamma | 7 (20%) | 15 (44%) |
| Low Ripple | 13 (38%) | 12 (35%) |
| High Ripple | 9 (26%) | 20 (58%) |
| Very High Ripple | 14 (%41) | 13 (38%) |
|  |  |  |

**Table 3. Proportion of regions with power differences between REM and wakefulness in the various bands.** The number of significant regions out of 34 possible regions for wakefulness differing from REM for each band. Only regions displaying a significant difference (p < 0.05) for both phasic and tonic REM being different than wakefulness are listed.


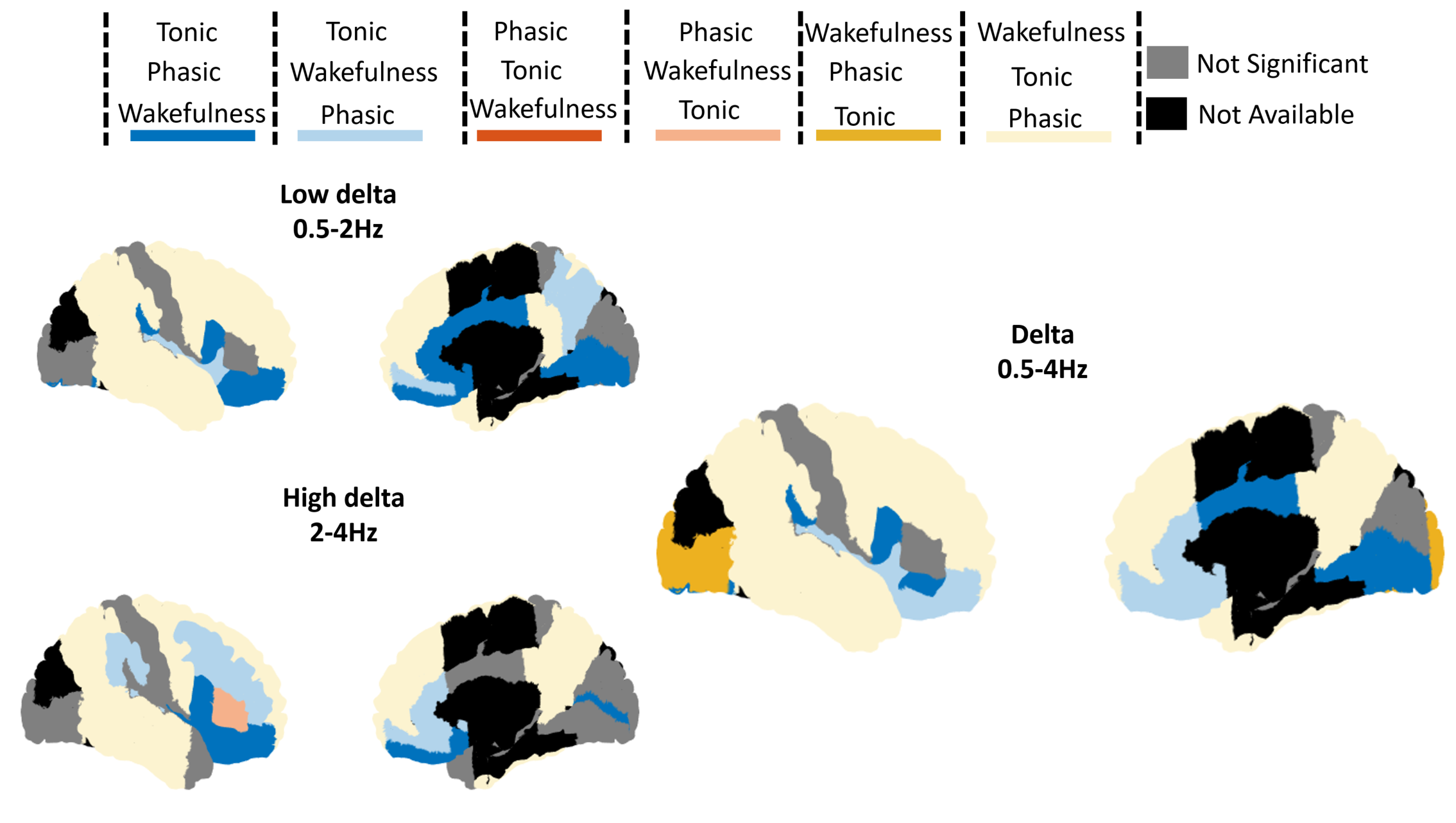


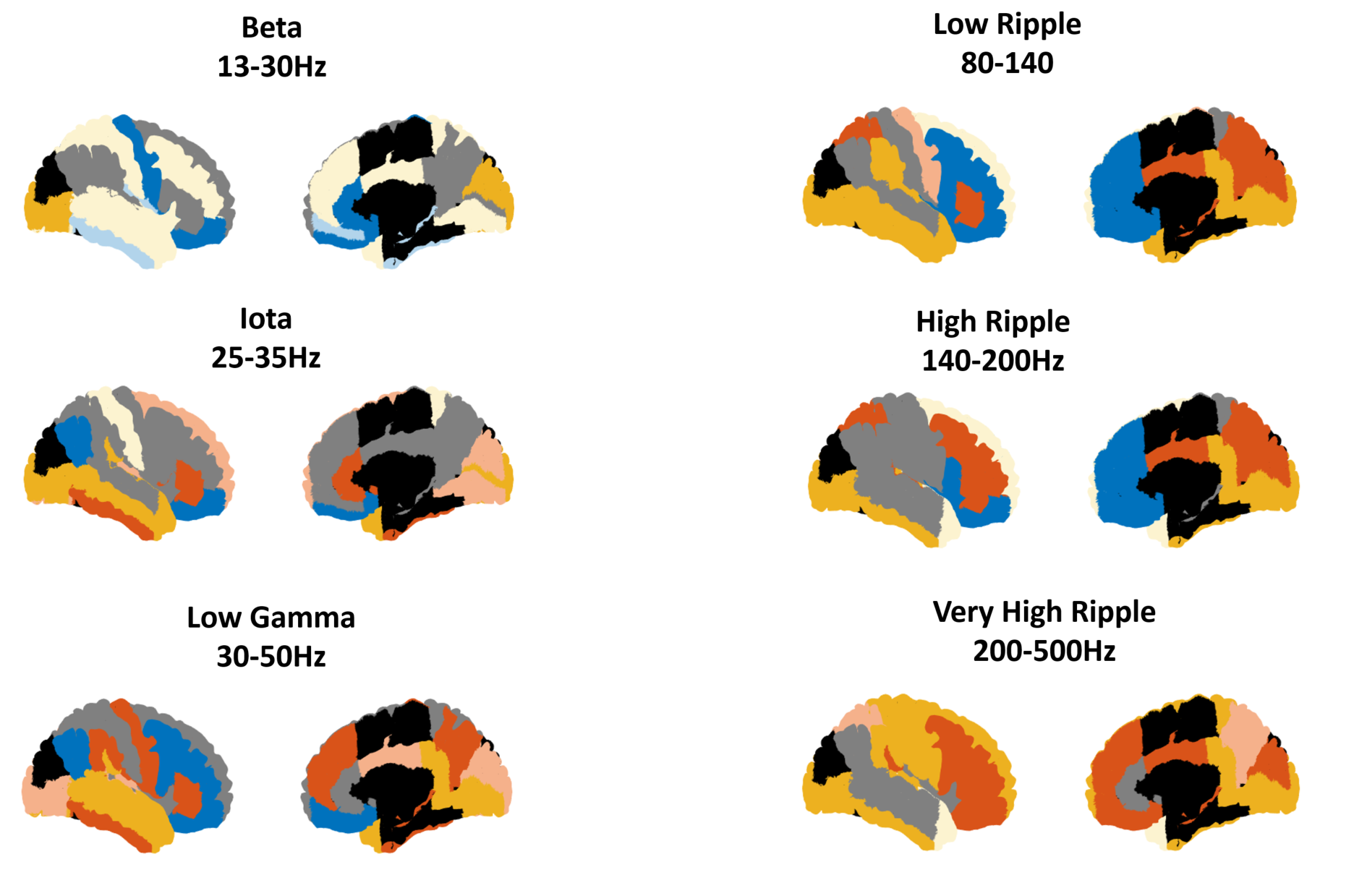


**Figure 3. Differences in power band trends between tonic REM, phasic REM, and wakefulness.** Depicted are the inner delta bands compared to the whole delta band, the Iota band next to the beta and low gamma bands, as well as the very high ripple bands next to the other ripple bands. The trends for significant differences between wakefulness and phasic and tonic REM are plotted for each power band. Every significant regional trend is represented with a color depending on which time period had the highest and lowest power. For example, red represents regions where phasic REM had the highest power, followed by tonic REM, and then wakefulness.


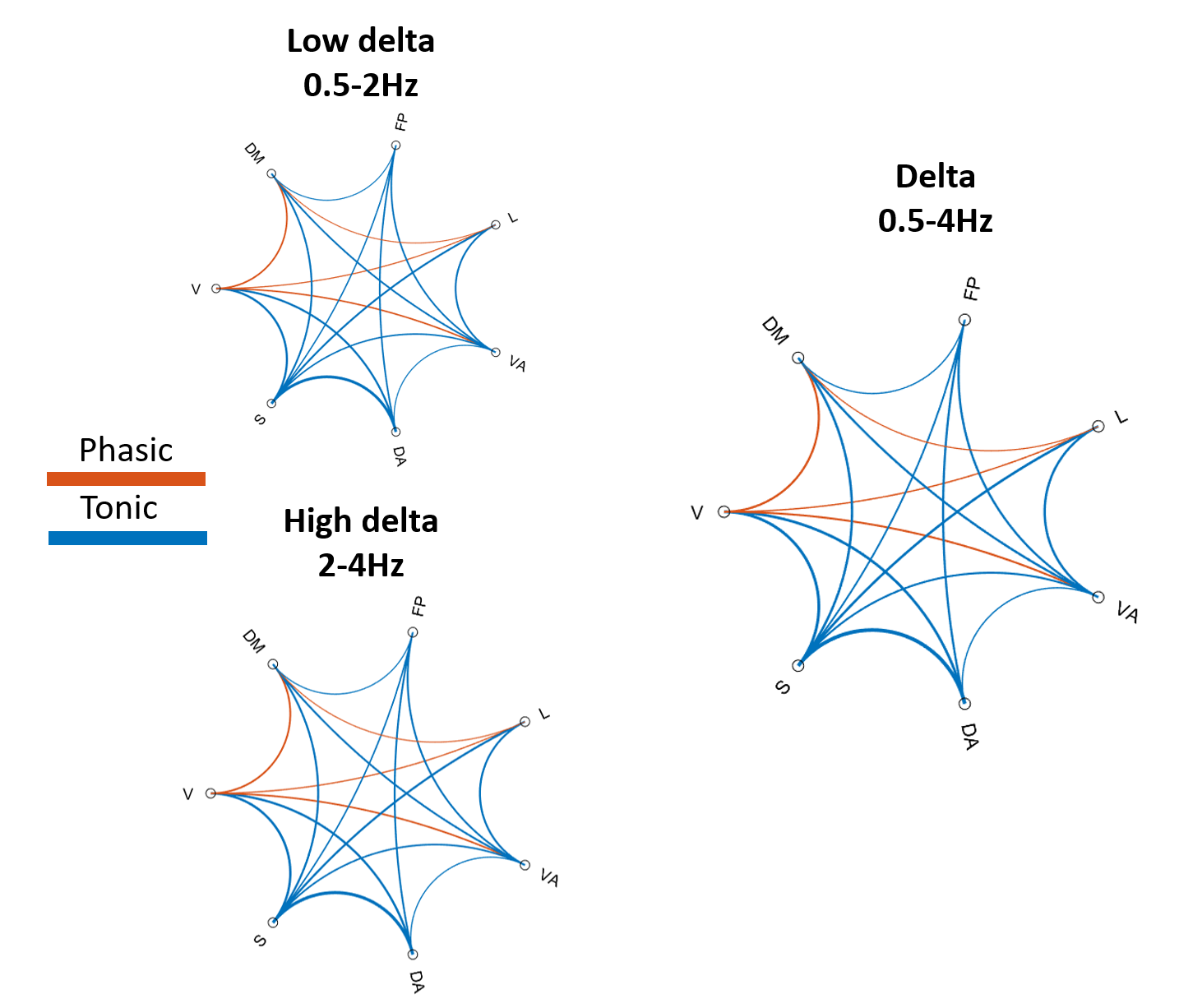


**Figure 4. Differences in delta band network-based connectivity between phasic and tonic REM.** Depicted are the lower and higher delta bands compared to the whole delta band. Effect sizes (Cohen's d) are plotted as the size and color of connecting lines between each network pair with significant differences between matching time periods of phasic and tonic REM. Significance was set to 0.05 after false discovery rate correction. V - Visual, S - Somatomotor, DA - Dorsal attention, VA - Ventral attention, L - Limbic, FP - Frontoparietal, DM – Default mode network.


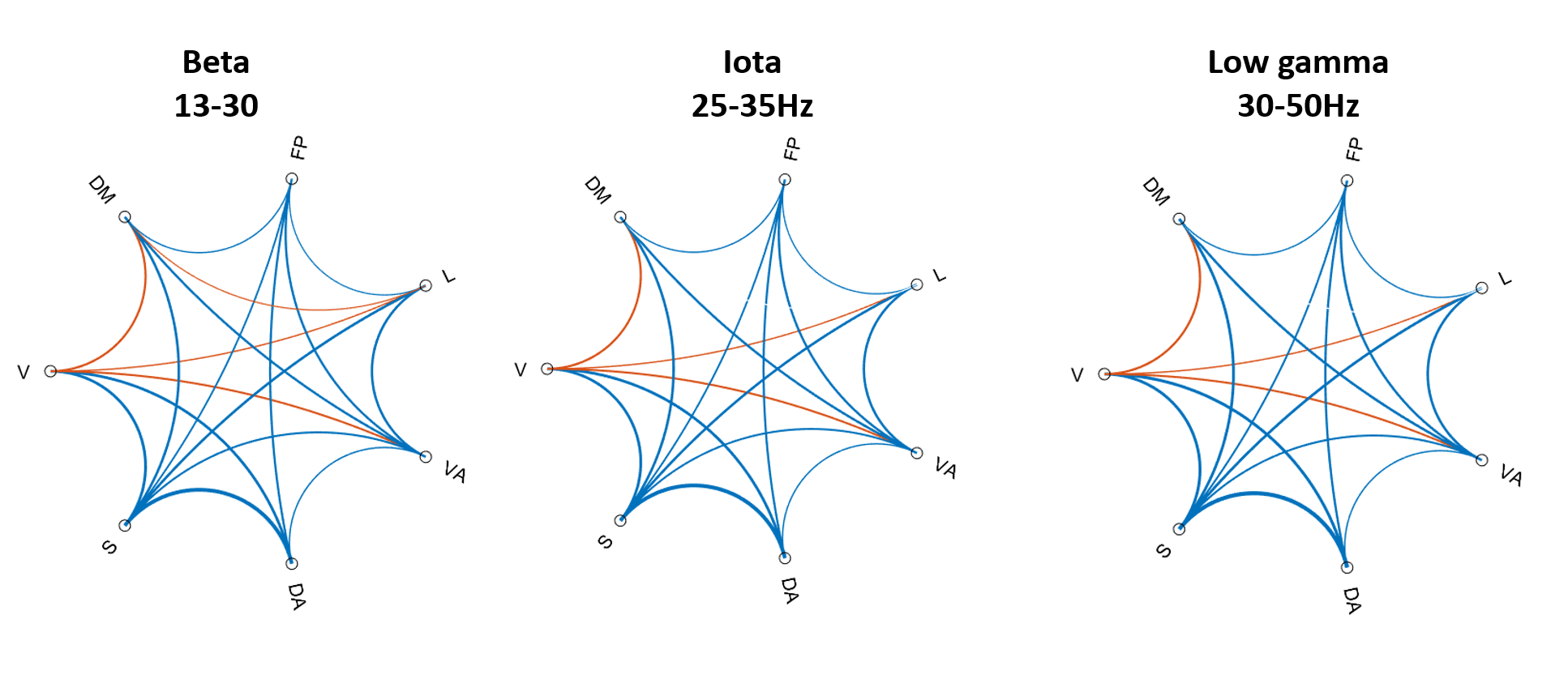


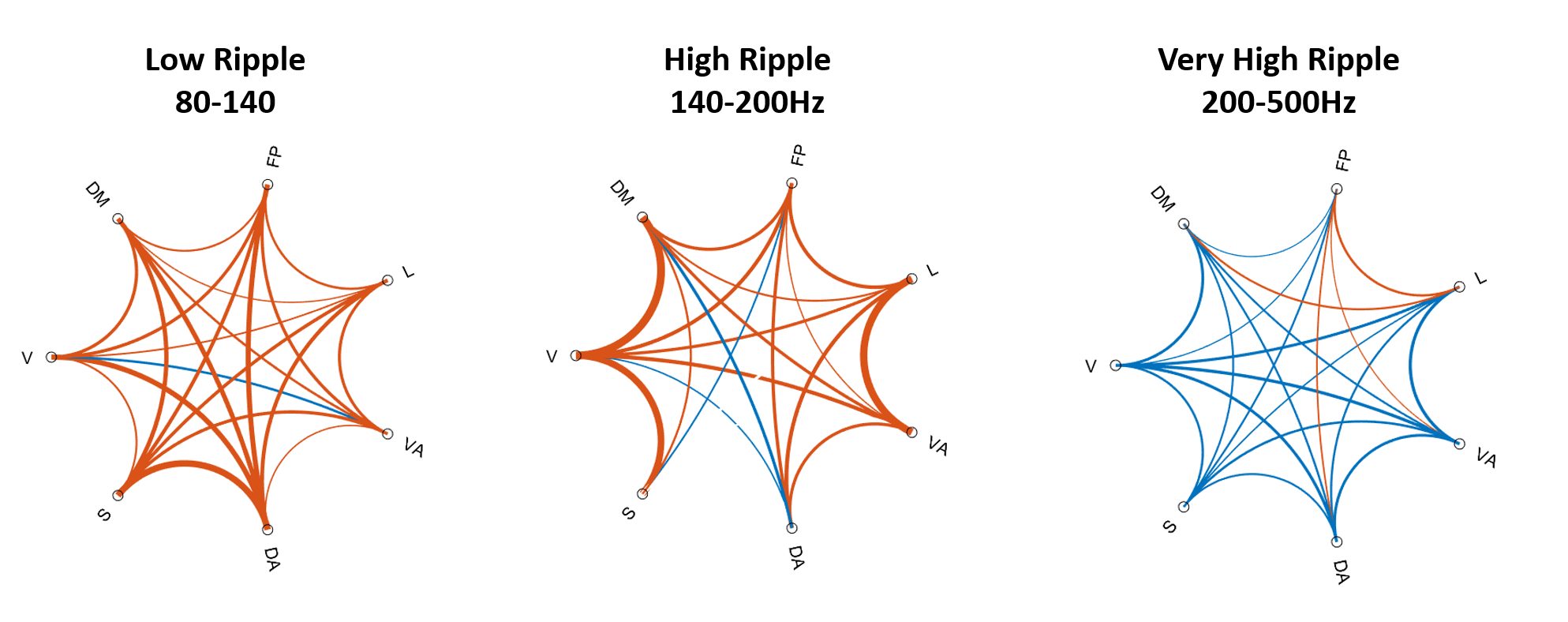


**Figure 5. Differences in delta band network-based connectivity between phasic and tonic REM.** Depicted are high frequency bands. Effect sizes (Cohen's d) are plotted as the size and color of connecting lines between each network pair with significant differences between matching time periods of phasic and tonic REM. Significance was set to 0.05 after false discovery rate correction. V - Visual, S - Somatomotor, DA - Dorsal attention, VA - Ventral attention, L - Limbic, FP - Frontoparietal, DM – Default mode network.
